# Management of subjects with Intermittent Explosive Disorder and Autism Spectrum Disorder with Lumateperone

**DOI:** 10.1192/j.eurpsy.2023.1218

**Published:** 2023-07-19

**Authors:** N. Mehdiratta, S. Kalita, D. Birwatkar, A. Hirsch

**Affiliations:** 1 Psychiatry, Smell and Taste Treatment & Research Institute, Chicago, United States; 2 Psychiatry; 3 Spartan Health Sciences University, Vieux Fort, Saint Lucia

## Abstract

**Introduction:**

Use of lumateperone in reduction of aggression in patients with both autism spectrum disorder and intermittent explosive disorder has not heretofore been described.

**Objectives:**

Explore the impact of lumateperone treatment for intermittent explosive disorder in autism spectrum disorder.

**Methods:**

Case A: An illiterate non-verbal 18-year-old male, presented with history of behavior problems, developmental and intellectual delays. He attended special education programs at school where he had difficulty interacting with peers and teachers. For three weeks prior to presentation, he has displayed more anger and aggression, biting his hands, pounding on the walls and furniture, and screaming.

Case B: This 18-year-old male, presented with a history of hypsarrhythmia, Lennox-Gastaut syndrome, severe developmental delay, deficit in socializing with lack of interaction with others and autism, presented with aggression, agitation, and hostility, banging on walls and furniture and throwing objects. Coincident with this was an increase in frequency of myoclonic seizures occurring up to twenty-five seizures per day followed by a postictal period of shouting and screaming.

**Results:**

Case A: Psychiatric examination: Nonverbal, intermittently grunting and screaming, eyes darting, not responding to verbal commands. Posturing of arms in the air, in a hostile stance. Ten days post starting nightly lumateperone 42 mg, patient was no longer banging furniture nor biting. He remained non-verbal but without screaming and hostile behavior.

Case B: Psychiatric examination: Non-verbal screaming, throwing furniture, hitting walls and uncooperative to verbal commands. One month post starting lumateperone 42 mg nightly, while the seizures persisted, patient’s violent attacks were not severe. Two months later, the aggression was much less severe, that he could attend school. Behavioral problems with aggression would recur an hour before the next evening’s lumateperone dose.

**Conclusions:**

Lumateperone modulates a variety of neurotransmitters including glutamate, functions as a presynaptic partial dopamine agonist and postsynaptic dopamine antagonist, and enhances N-methyl-d-aspartate, an inhibitor of serotonin reuptake (Reddy, 2020), input, on any of these, may be its mechanism of action (Vyas, 2020). Perchance, its action as a 5HT-2A receptor antagonist may be its method of reducing aggression, as has been posited for its anti aggression effect in schizophrenia (Vyas, 2020). Lumateperone impacts on neurotransmitters, including substance P, which modulate aggression (Gretchen, 2020). Alternativity ASD correlated with dysfunction of Area VIIIA of the right posterior cerebellum and its connection with the left frontal cortex (Heemkerk, 2021). Lumateperone may act to stabilize these areas and thus inhibit aggression (Heemkerk, 2021). In those with aggression associated with ASD and IED, a trial of lumateperone may be worthwhile.

**Disclosure of Interest:**

None Declared

